# Limited utility of qPCR-based detection of tumor-specific circulating mRNAs in whole blood from clear cell renal cell carcinoma patients

**DOI:** 10.1186/s12894-019-0542-9

**Published:** 2020-02-04

**Authors:** Sinisa Simonovic, Christian Hinze, Kai M. Schmidt-Ott, Jonas Busch, Monika Jung, Klaus Jung, Anja Rabien

**Affiliations:** 1 Department of Urology, Charité - Universitätsmedizin Berlin, corporate member of Freie Universität Berlin, Humboldt-Universität zu Berlin, and Berlin Institute of Health, Berlin, Germany; 2 Berlin Institute for Urologic Research, Berlin, Germany; 3 Max-Delbrück-Center for Molecular Medicine (MDC), Berlin, Germany; 4 Department of Nephrology and Medical Intensive Care, Charité - Universitätsmedizin Berlin, Berlin, Germany

**Keywords:** Renal cell carcinoma, CDK18, CCND1, Biomarker, Blood, PAXgene

## Abstract

**Background:**

RNA sequencing data is providing abundant information about the levels of dysregulation of genes in various tumors. These data, as well as data based on older microarray technologies have enabled the identification of many genes which are upregulated in clear cell renal cell carcinoma (ccRCC) compared to matched normal tissue. Here we use RNA sequencing data in order to construct a panel of highly overexpressed genes in ccRCC so as to evaluate their RNA levels in whole blood and determine any diagnostic potential of these levels for renal cell carcinoma patients.

**Methods:**

A bioinformatics analysis with Python was performed using TCGA, GEO and other databases to identify genes which are upregulated in ccRCC while being absent in the blood of healthy individuals. Quantitative Real Time PCR (RT-qPCR) was subsequently used to measure the levels of candidate genes in whole blood (PAX gene) of 16 ccRCC patients versus 11 healthy individuals. PCR results were processed in qBase and GraphPadPrism and statistics was done with Mann-Whitney U test.

**Results:**

While most analyzed genes were either undetectable or did not show any dysregulated expression, two genes, CDK18 and CCND1, were paradoxically downregulated in the blood of ccRCC patients compared to healthy controls. Furthermore, LOX showed a tendency towards upregulation in metastatic ccRCC samples compared to non-metastatic.

**Conclusions:**

This analysis illustrates the difficulty of detecting tumor regulated genes in blood and the possible influence of interference from expression in blood cells even for genes conditionally absent in normal blood. Testing in plasma samples indicated that tumor specific mRNAs were not detectable. While CDK18, CCND1 and LOX mRNAs might carry biomarker potential, this would require validation in an independent, larger patient cohort.

## Background

In the United States, it is estimated that 65,340 new cases and 14,970 deaths from kidney cancer will occur in 2018 [1]. ccRCC is the most common renal malignancy, accounting for around 80% of the cases [2]. Together with papillary and chromophobe carcinoma, it comprises 2% of all cancers worldwide [3]. Renal cell carcinoma (RCC) incidence increases markedly with age, peaking at 50–70 years, with males being affected twice more frequently than females [4]. The major risk factors for RCC include excess body weight, hypertension and cigarette smoking [5] and associations have also been made with different lifestyle, dietary, occupational and environmental factors [6]. Primary RCC displays no early clinical symptoms as most renal masses remain asymptomatic until the late stages of the disease, with over 50% of all cases of RCC being discovered by chance during imaging studies for other comorbidities [7, 8]. Only 10% of patients have the classical triad of symptoms: hematuria, flank pain and weight loss. Around 25% of RCCs have already metastasized by the time of diagnosis [9]. RCC is mostly unresponsive to conventional chemotherapy and radiation, which is the main reason for treatment failures [10, 11]. The gold standard for the management of renal masses is nephrectomy, in spite of which approximately 30% of patients develop recurrence or metastases [12, 13], which require systemic therapies and are associated with high mortality.

As current prognostic models based on conventional clinicopathological and imaging data have limited accuracy, new biomarkers are needed for early detection, improved diagnostics and the prediction of the clinical outcome of patients with RCC [14–17]. The ideal biomarker or biomarker panel should have high specificity, sensitivity, and reproducibility. Plasma, serum, and urine have recently gained interest in the field of cancer biomarker discovery. They represent potential sources of valuable biomarkers, containing proteins, DNA, and various RNA species, with blood being especially suitable in terms of kidney disease and low invasiveness. Steady progress in the field is being made, however to date, none of the identified ccRCC biomarkers have been clinically validated [18].

RNA circulating in blood is highly degraded (usually less than 100 bp in length [19]) and even after the introduction of systems that enable the stabilization and storage of whole blood mRNA (e.g. the PAXgene platform) studies tend to be limited to shorter RNA subspecies, or those protected from degradation due to their specific structure or association with proteins or membranous vesicular structures such as exosomes. Analyzing ccRCC biomarkers in urine would be particularly convenient, however this field is far less fruitful compared to blood studies. Similarly as for blood, urine is problematic in terms of RNA detection because of the presence of RNAses, but also because of PCR inhibition [20], which is steering the focus into the analysis of shorter RNA subspecies. Especially in the case of microRNA (miRNA), liquid biopsy has expanded from use in plasma to other bodily fluids in an increasing number of malignancies, making rapid progress since 2008 [21]. Along with miRNA, the use of Circulating Tumor Cells (CTCs), cell free DNA (cfDNA), and more recently circular RNA (circRNA) and long non-coding RNAs (lncRNA), are proving to be far more viable strategies, as for most tumors there is somewhat sporadic progress in detecting tumor-derived mRNA in blood and associating it with cancer prognosis, for example as described here [22–24]. A further issue that complicates tumor-derived RNA detection in blood is the uncertainty regarding the exact RNA origin, i.e. whether it is derived from solid tumor or CTCs, and what percentage of source cells are living and actively secreting RNA as opposed to undergoing apoptosis [25].

The first of the five proposed stages in biomarker development is the comparison of tumor with nontumor tissue [26]. Here techniques such as microarrays and more recently RNAseq are used to assess gene expression, while protein expression profiles are based on immunohistochemistry and mass spectroscopy, with the goal of discovering genes displaying dysregulation (usually overexpression in tumor compared to normal tissue). This phase is followed by the development of a clinical assay utilizing blood for non-invasive screening. Of course, the blood levels of selected genes do not have to precicely mirror the expression in tissue, as a result of e.g. the specific rate of mRNA release from cancer tissue into blood. The approach of using mRNA expression of tumor tissue as a starting point and analyzing the levels od respective transcripts in blood by RT-qPCR has been previously used with success, resulting in promising assays deserving of clinical validation. A recent study showed the validation of an RT-PCR assay based on prostate-specific RNA in whole blood from patients with metastatic castration-resistant prostate cancer (mCRPC) [27]. Several databases were consulted to select a panel of genes that were overexpressed in prostate tissue but showed no detection in peripheral blood mononuclear cells (PBMC). This was followed by RT-PCR analysis of blood samples of cancer patients and volunteers, resulting in the establishment of a 5-gene panel that enhances and complements the previously established CTC enumeraration assay. Similarly, in another study focusing on early detection of colorectal cancer [28], meta-analysis of microarray data was used to identify RNAs with highest diffeential expression between cancer tissue and normal blood samples. Subsequent RT-qPCR analysis revealed that blood expression of 3 specific genes shows promising sensitivity and specificity with respect to detection of this cancer.

In this study, TCGA database was used as a starting point to identify genes which are most highly overexpressed in the tissue of ccRCC patients, after which a subset containing genes that according to other databases have no blood expression was evaluated by qPCR in whole blood samples from ccRCC patients and healthy individuals. While RNA transcripts of some of these genes had good detectability in blood, none of the genes were significantly up-regulated in blood from ccRCC patients and two genes paradoxically displayed downregulation.

## Methods

### Patients and samples

The staging and grading of the tumor samples were done according to the 2002 TNM classification and the Fuhrman grading system [29, 30]. The ccRCC tissue samples were obtained during partial or radical nephrectomy at the University Hospital Charité in Berlin in 2011 and blood samples in the period between 2010 and 2016. Tissue samples were frozen in liquid nitrogen directly after surgical resection and stored at − 80 °C until RNA extraction. They came from tumor and matched normal tissue of 3 male patients without diagnosed metastasis (ages: 47–71; tumor stages: 2 x pT1, and pT3; grading: G1, G2, G3). PAXgene blood samples were obtained from 27 individuals and included 16 ccRCC samples, out of which 10 were non-metastatic (8 male and 2 female patients; median age 70, range 47–84 years; tumor staging: 1x pT1, 2x pT2, 7x pT3; grading: 2x G1, 7x G2, 1x G3) and 6 metastatic: (5 male and 1 female patients; median age 67, range 47–72 years; tumor staging: 6x pT3; grading: 5x G2, 1x G3). In total there were 11 samples without diagnosed cancer, 4 from patients suffering from non-cancer kidney illnesses, and 7 healthy volunteers (7 male and 4 female; median age 47, range 29–80 years).

### Bioinformatics analysis

**Fig. 1:**
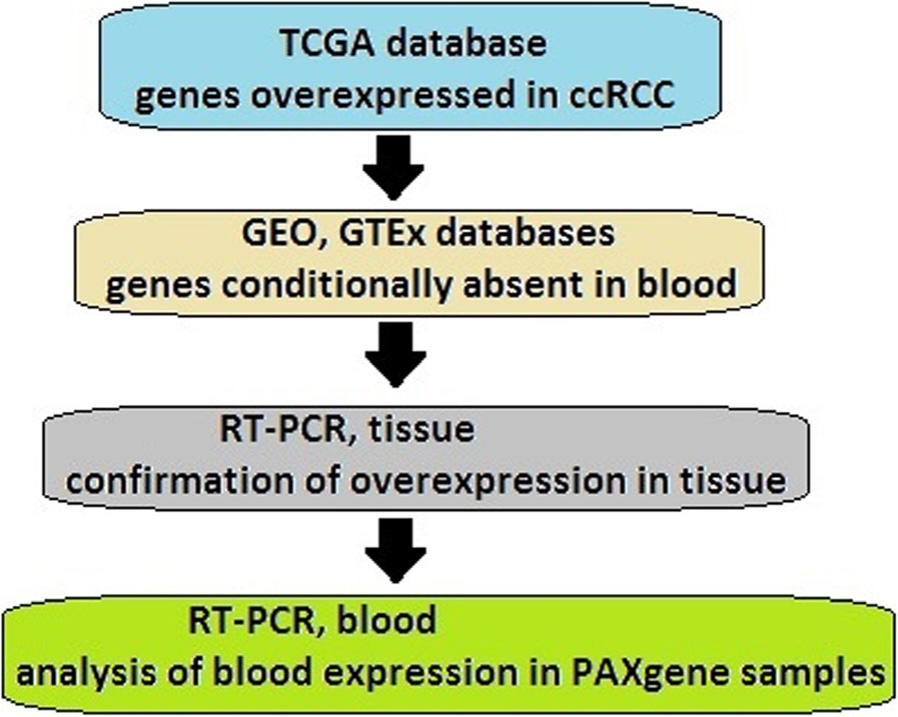
Workflow diagram

The first stage in gene selection was the analysis of ccRCC expression in TCGA database, followed by the use of GEO and GTEx databases to remove genes present in blood (Fig. 1). Subsequently, in order to evaluate candidate genes with respect to their suitability to serve as blood biomarkers by distinguishing ccRCC vs. normal patients, their expression was first tested by RT-qPCR in ccRCC and normal tissues, and secondly in blood samples of cancer patients versus non-cancer patients and healthy donors. According to the bioinformatics analysis, the higher expression in ccRCC tissue would be expected to be confirmed as compared to normal tissue, and subsequently, when PAXgene blood samples are tested, the higher expression of at least some of the candidate genes would hopefully be determined in PAX blood from cancer patients compared to healthy. In order to obtain RNA seq based expression profiles in ccRCC and compare them to normal tissue as well as blood, the Cancer Genome Atlas database (TCGA, [31]) was used. The TCGA data portal is the largest and most commonly used public resource providing somatic and germline mutation, gene expression, gene methylation and copy number variation (CNV) data sets, amongst others, for several thousands of tumor samples. Data was obtained for 470 ccRCC patients, including 68 samples from matched normal tissue. In cases where multiple samples corresponded to a single patient, average expression values were calculated. Out of 20,533 TCGA genes in total, blood expression data from sources described below was found for 20,466 genes. Ideally candidate genes should not have wide expression domains; so as to provide a measure of kidney specificity for a gene, Tissue-specific Gene Expression and Regulation database (TiGER, [32]) was consulted, which is based on the analysis of the NCBI EST database [33] for 30 human tissues and contains tissue-specific expression profiles for 20,000 UniGenes. From 458 enriched in kidney, genes also expressed in blood, liver, prostate and bladder were deducted, leaving a list of 95 conditionally named ‘kidney specific’ genes.

In order to obtain blood expression profiles, a comprehensive search for RNA seq expression data from healthy individuals was made in literature and online databases. Gene Expression Omnibus database (GEO, [34]) archives and freely distributes microarray, next-generation sequencing, and other forms of high-throughput functional genomics data. This database was searched by variations of ‘blood [Sample Source] AND *Homo sapiens* [Organism] AND high throughput sequencing [Platform Technology Type]’ providing a total of seven usable datasets altogether comprising 91 individual blood samples. Further 376 blood samples were obtained from GTEx database [35] and an additional source of one blood sample pooled from five individuals was kindly provided by Dr. Zhao and Dr. Zhang of Pfizer.

In order for expression profiles in important organs or organs related to the urological system to be given some relevance, RNA seq data from normal tissue was also considered in the analysis. From TCGA database, data was obtained for normal liver and bladder (9 and 11 samples respectively) and an analogous GEO search resulted in a recovery of a small number of samples for kidney, liver and bladder. Additional samples for kidney and liver (pooled from multiple donors) were included from RNA seq Atlas [36] (Table 1).

**Table 1:** Sources of expression profile datasets

Database	Tissue	Sample number	Source
GEO/GSE53655	blood	6	whole blood/PAXgene
GEO/GSE72509	blood	18	whole blood/PAXgene
GEO/GSE51799	blood	6	whole blood/PAXgene
GEO/GSE51799	blood	16	peripheral blood mononuclear cells
GEO/GSM833103	blood	16	whole blood/PAXgene
GEO/GSM1647922, Personal correspondence	blood	12	whole blood/EDTA, Tempus
Personal correspondence, Pfizer [37]	blood	1, pooled from 5	whole blood/PAXgene
GTEx	blood	376	whole blood
TCGA	liver, normal matched	9	cancer patients
TCGA	bladder, normal matched	11	cancer patients
GEO/GSE69360	kidney	2	adult normal tissue
GEO/GSE69360	liver	2	adult normal tissue
RNA seq Atlas	kidney	1, pooled	normal tissue
RNA seq Atlas	liver	1, pooled	normal tissue
GEO/GSE35178	bladder	1	adult normal tissue

Processing of data downloaded from TiGER database, RNA seq expression data, calculation of rpkm values (reads per kilobase million) where necessary, translation of gene names and statistics were done in Python. Rpkm values were calculated according to the formula: raw count × 1,000,000/(gene length x library size). Translation of gene names was done using BioMart [38]. Mann-Whitey U test was used to discern between cancer and matched normal samples from TCGA with statistical significance defined as *p* < 0.05. In cases of gene expression entries with multiple isoforms, replicate samples, duplicate gene names, absolute highest values were taken, so as not to underestimate the possible presence in blood.

### RNA isolation and RT-qPCR analysis

Total tissue RNA (1 μg) was purified using miRNeasy Kit (Qiagen, Hilden, Germany) following homogenization using TissueLyser II (Qiagen). Total RNA from PAXgene blood tubes was purified using the PAXgene Blood miRNA Kit (Qiagen). Total RNA concentration was determined by NanoDrop 1000 Spectrometer (Thermo Fisher Scientific Inc., Wilmington, DE, USA) by measuring the absorbance at 260 nm and RNA purity by measuring A260/280 ratios. The integrity and size distribution of the tissue and blood derived RNA was analyzed using Bioanalyzer (Agilent RNA 6000 Nano Kit). Only samples with RNA integrity number values equal or above 7 were included. RNA samples from normal tissues were pooled together and the same was done with the ones from cancer, producing one normal pool (NN) and one cancer pool (NC). Complementary DNA synthesis was performed using the Transcriptor First Strand cDNA Synthesis Kit (Roche Applied Science, Mannheim, Germany) with a mix of random hexamer and anchored-oligo(dT) primers. RNA was also isolated and transcribed from the renal cell carcinoma cell line 786–0 to assess the quality of all newly made cDNA from tissue and PAXgene blood samples. Normalization of the RT-qPCR data was done using the kidney reference gene peptidylproline isomerase A (PPIA) [39].

Primers were designed for SYBR Green using NCBI’s PrimerBlast and Primer3 (see Additional file 1), so as to cover the maximum number of isoforms. The criteria for primer design were: amplicon length 60–150 nt, primer length 18–30 nt, intron spanning (intron length > 1000 nt), GC content 40–60%. For certain genes UPL probes were used in which case primers were automatically suggested with a given probe by the online tool (Universal Probe Library, Roche [40]), and common assays were selected for genes with multiple isoforms.

The relative quantification of transcripts was done on the Light Cycler 480 (Roche) using the QuantiTec SYBR Green PCR Kit (Qiagen) as previously described [37]. In case of UPL probes LightCycler 480 Probes Master Kit (Roche) was used. PCR was done on 96-well plates, with kidney cancer cell line 786–0 and ccRCC tissues as positive controls. PCR conditions were optimized where necessary and the size of PCR products was confirmed by electrophoresis using Bioanalyzer (Agilent DNA 1000 Kit). PCR data were analyzed by qBasePLUS software (Biogazelle NV, Gent, Belgium). With respect to the qBasePLUS processing, the samples were divided in 2 or 3 groups: normal vs. all cancer samples, i.e. cancer and metastatic cancer in a single group, as shown in the table ‘qBasePLUS results: normal vs. all cancer samples’ (see Additional file 2), normal vs. non-metastatic cancer, normal vs. metastatic cancer, and non-metastatic cancer vs. metastatic, as shown in the table ‘qBasePLUS results: non-metastatic cancer vs. metastatic samples’ (see Additional file 3). Results were calculated for 100% PCR efficiency and ´unpaired´ experimental design.

### Statistics

Statistical analysis was done with GraphPad Prism 6.07 (GraphPad Software, San Diego, CA, USA) and qBasePLUS, using the Mann-Whitney U-test. *P* values < 0.05 were considered statistically significant. Graphs were *generated in GraphPad Prism using the* Mann-Whitney U-test.

## Results

### Candidate gene selection

In order to obtain a list of genes potentially useful as biomarkers, only genes with supposedly no blood expression, favorable statistical distance between distributions of cancer and normal values and high expression in cancer were taken into account. Regarding blood expression, values below 1 rpkm were considered low enough as to signify possible non expression, with respect to the sensitivity of detection. As a measure of distance of cancer and matched normal tissue distributions, the ratio of 5th percentile of cancer distribution with 95th percentile from normal was taken, and values above 0.5 considered favorable. Another measure of distance was calculated where the score represents the multiplication of probabilities of patients from each distribution falling within the overlap interval (score = Xprob x Yprob). Individual probabilities are calculated as the number of patients whose rpkm values fall within the overlap interval, divided by the total number of patients in the distribution (Xprob = patients within the overlap interval/total number of patients). Score is assigned 0 if the distributions do not overlap, and 1 for identical distributions. In cases when one distribution is within the other, but there are no patients from the larger one which fall into the overlap interval (they are distributed on both sides of it) score is assigned 1 as those genes are not valuable for further analysis. This method of calculating statistical distance is generally stricter then the percentile ratio, with favorable distance represented by values less than 0.3.

For genes of interest, the expression levels in liver, bladder, prostate and kidney in healthy individuals were also taken into account, giving preferential ranking to genes with lower rpkm values. Literature, the Human Protein Atlas [41], and OMIM [42] were consulted to gather information regarding gene function and expression domains of selected genes. Gene functions related to metabolic pathways in kidney or implicated in cancer (especially genes linked to ccRCC and hypoxia-inducible factors HIF1α and HIF2α) together with absence of expression in bone marrow and immune system, low or no expression in most tissues, and enrichment in kidney were considered favorable with respect to gene ranking.

A group of 20 genes were found to strictly fulfill expression criteria (defined as: blood expression GEO sources 95th percentile <1rpkm, GTEx 95th percentile <=1; fold change TCGA cancer median/matched normal tissue median > 1; distribution distance 5th percentile TCGA cancer/95th percentile matched normal tissue > 0.5, TCGA cancer median > 5rpkm) (Table 2, first 20 genes). The first 13 genes have median cancer values above 10. In addition, when it is considered that the rate of release of RNA from ccRCC into blood may be much higher than from normal kidney, as well as the presence of circulating tumor cells, the fold change median cancer/matched normal tissue, as well as the percentile ratio distribution distance measure become less relevant and may be relaxed in terms of gene selection. A similar argument follows for blood expression considering that individual blood sources may not be fully reliable and false outliers may be present. This enables the inclusion of certain genes which do not satisfy all the criteria fully, but may excel in some (last 11 genes in the table).

**Table 2:** Candidate genes

GENE	MEDIAN RPKM in ccRCC (based on expression data from TCGA consortium, [31])	FOLD DIFFERENCE, median rpkm in ccRCC vs. median rpkm in normal kidney (based on expression data from TCGA consortium, [31])	RPKM DISTRIBUTION DISTANCE, 5th percentile ccRCC/ 95th percentile normal kidney (based on expression data from TCGA consortium, [31])	BLOOD RPKM value of 95th percentile (based on GEO database [34])	BLOOD RPKM value of 95th percentile (based on GTEx database [35])
NDUFA4L2	701	145	1.06	0.16	0.15
EGLN3	174	23.2	0.93	0.72	0.62
CA9	117	1218	3.74	1	0.13
CCND1	138	4.34	0.58	0.58	0.37
CAV2	110	3.98	0.69	0.43	0.43
ESM1	92.5	12.4	0.6	0.77	0.2
PPP1R3C	20.1	3.66	0.54	0.03	0.67
STC2	19.1	22.3	1.33	0	0.07
NPTX2	18.4	150	1.48	0.07	0.13
ANGPT2	17.1	10.4	0.54	0.11	0.08
DGCR5	15.2	25.9	0.99	0.3	0.17
DOCK6	13.2	2.08	0.51	0.26	0.68
FABP6	11.9	91.3	1.18	0.62	0.85
TMEM133	8.9	2.67	0.52	0.60	0.24
LZTS1	8.81	5.54	0.52	0.26	0.25
COX4I2	6.85	4.37	0.52	0.06	0.15
KIAA1274	6.71	4.11	0.58	0.78	0.41
LPIN3	6.14	2.29	0.54	0.12	0.17
FKBP9L	6.11	1.32	0.51	0.71	0.14
RAB42	5.90	5.47	1.02	0.17	0.49
MET	112	2.08	0.36	0.07	0.1
CDK18	85.4	5.29	0.28	1.21	0.85
CP	79.7	21.3	0.11	0.89	0.4
TMEM45A	62.9	2.43	0.71	0.13	2.31
LOX	51.0	10.8	0.22	0.22	0.29
GAL3ST1	49.4	8.14	0.3	0.15	0.11
CYP2J2	49	39.3	0.27	0.65	0.2
NOL3	44.1	10.6	1.36	1.98	2.47
FBXO17	38.7	2.77	0.5	2	0.12
FABP7	19.9	974	0.22	0	0.05
BARX2	19.1	6.01	0.27	0.42	0.04

Many of these genes have previously been implicated in ccRCC, largely in micro-array studies [43–50]. This analysis identified this group of genes as having zero or low RNA blood presence, suggesting their potential use as ccRCC biomarkers in blood.

### Analysis of the expression of candidate genes

**Fig. 2:**
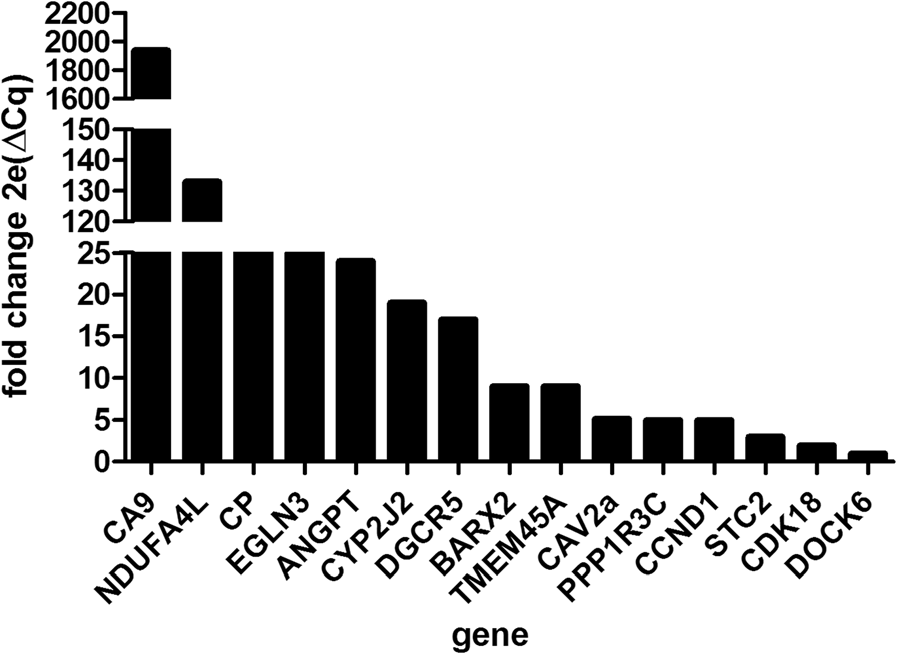
Confirmation of TCGA data by RT-qPCR: Candidate genes were overexpressed in ccRcc compared to normal tissue (all values are above 1). Fold change is calculated as 2exp (Cqnormal-Cqcancer)

To obtain an approximate overview of the levels in tissue, the expression was analyzed in cancer versus normal tissue for 15 genes of highest interest and the bioinformatics analysis was confirmed, as all these genes showed increased levels in cancer, notably so CA9 and NDUFA4L2 (Fig. 2). Certain genes were excluded from the analysis because of detection issues (multiple isoforms, etc.).

Blood testing consisted of two stages: in the first stage 3 PAXgene cancer samples were used (Table 3), and only genes with good (Cq < 33) detectability were selected for the second stage of blood testing with 24 more PAXgene samples (13 cancer and 11 normal/healthy), in order to broadly evaluate their expression. Especially good detectability was shown for following genes: CDK18 (Cq = 27), EGLN3 (Cq = 26), TMEM45A (Cq = 28), CAV2 (Cq = 26). A larger number of genes was undetectable or with extremely high Cq values. The genes with the supposedly highest potential based on bioinformatics and tissue PCR analysis (NDUFA4L2 and CA9) had low detectability with very high Cq values (around 34). Nevertheless, NDUFA4L2 was tested on all 27 samples and was confirmed to be undetectable. In summary, 9 genes were finally selected for the second stage of testing (CAV2a, FABP7, ESM1, NOL3, LOX, CDK18, EGLN3, TMEM45A, CCND1). In the second stage, the expression levels turned out to be similar in cancer versus normal blood for most genes, except for CDK18 and CCND1 which paradoxically turned out to be downregulated in cancer blood (Table 4). Additional testing with 10 plasma samples indicated non-measurable expression. There was no correlation between the expression levels in blood for CDK18, CCND1, and LOX, and patient data such as age, tumor grade and stage.

**Table 3:** Evaluation of candidate genes by RT-qPCR in tissue and 3 blood samples

Candidate genes	Confirmed Higher Expression In Ccrcc Vs Normal Tissue; fold change cancer/normal	Detectability In 3 PAX blood ccRCC SAMPLES, mean Cq value
NDUFA4L2	yes; 133	> 33
EGLN3	yes; 25	26.1
CA9	yes; 1938	> 33
CCND1	yes; 5	31.5
CAV2	not tested	26.1
ESM1	not tested	30.6
PPP1R3C	yes; 5	> 33
STC2	yes; 3	> 33
NPTX2	yes; 24	> 33
ANGPT2	yes; 24	> 33
DOCK6	yes; 1	> 33
FABP6	not tested	> 33
MET	not tested	> 33
CDK18	yes; 2	27.6
CP	yes; 40	> 33
TMEM45A	yes; 9	28.8
LOX	not tested	30.7
GAL3ST1	not tested	> 33
CYP2J2	yes; 19	> 33
NOL3	not tested	29.8
FABP7	not tested	32.5
BARX2	yes; 9	> 33

**Table 4:** Evaluation of expression in the second stage of blood testing with 27 samples

Candidate genes	Fold change cancer/normal	*P* value	Significantly different expression of ccRCC vs. normal in 27 pax blood samples
EGLN3	−1.26	0.215	No
CCND1	−1.55	0.039	Downregulated in ccRCC
CAV2	1.16	0.497	No
ESM1	−1.21	0.668	No
CDK18	−2.10	0.001	Downregulated in ccRCC
TMEM45A	−1.04	0.734	No
LOX	−1.31	0.668	No, tendency towards upregulation in metastatic ccRCC
NOL3	−1.05	0.641	No
FABP7	−1.13	0.671	No

**Fig. 3:**
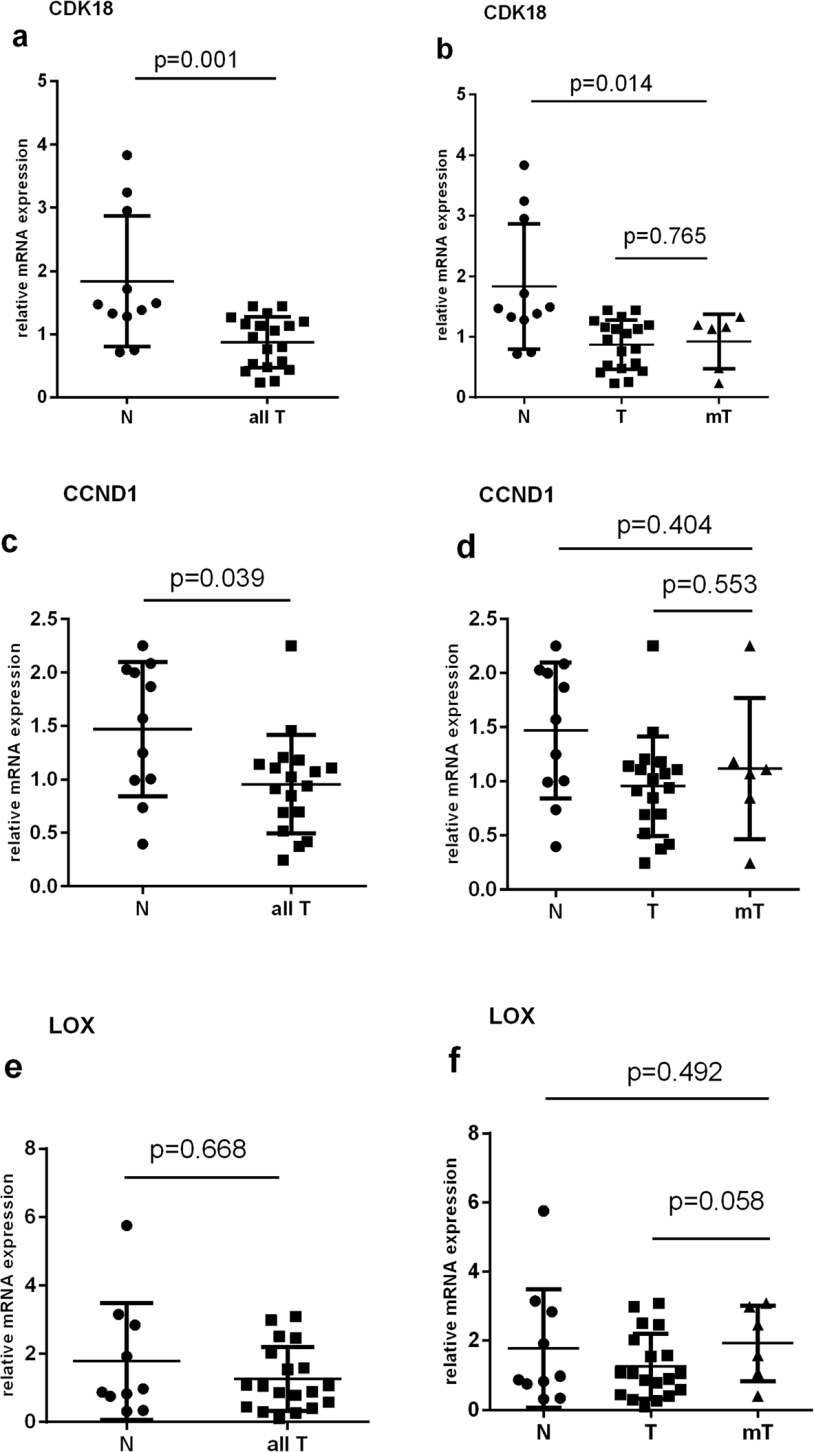
Blood relative mRNA expression of CDK18, CCND1, and LOX based on the qBase exported relative quantity (RQ) values, calculated from Cq values, according to the formula: RQ = 2 ^(meanCq-Cq)^; results from qBase (RQ values) were processed in *GraphPad Prism in order to generate graphs using the* Mann-Whitney U-test. N- normal patient samples; T- tumor patient samples; mT- metastatic. **a** CDK18 was underexpressed in PAX blood tumor samples compared to normal PAX blood. **b** There is no significant difference in expression of CDK18 between tumor and metastatic tumor PAX blood samples. **c** CCND1 was underexpressed in PAX blood tumor samples compared to normal PAX blood. **d** There is no significant difference in expression of CCND1 between tumor and metastatic tumor PAX blood samples. **e** There is no significant difference in expression of LOX in PAX blood tumor samples compared to normal PAX blood. **f** Lox shows a tendency towards upregulation in metastatic compared to non-metastatic tumor PAX blood samples

The downregulation of CDK18 RNA in cancer blood (metastatic and non-metastatic grouped together) compared to normal was significant with *p* value = 0.001, whereas CCND1 was downregulated with *p* = 0.039 (Fig. 3). For both genes, there was no significant difference in levels when non-metastatic and metastatic samples were compared with each other. The results also showed a tendency towards upregulation for LOX when non-metastatic were compared to metastatic cancer samples, with the *p* value very close to significant (*p* = 0.058) (Fig. 3).

## Discussion

In this study, a gene panel was made comprising the most highly overexpressed genes in ccRCC tissue whose mRNA also had the potential of being absent in the blood of healthy individuals. The first stage in the construction of this panel was the TCGA database- to select a panel of the most overexpressed genes in ccRCC, followed by the GEO and GTEx databases- to deduct from this panel genes showing measurable expression in the blood of healthy individuals. After confirming the tissue overexpression on samples from ccRCC patients in the next step, RT-qPCR analysis was done to evaluate mRNA levels in whole blood of ccRCC patients vs. patients without ccRCC and healthy donors. Measurable genes did not show overexpression in normal blood while two genes exhibited downregulation.

### Whole blood analysis of selected genes does not show increased mRNA levels

The genes that had highest potential based on the bioinformatics analysis were NDUFA4L2 and CA9. According to the TCGA, the former has very high median expression in ccRCC tissue (701 rpkm) whereas the latter has the highest overexpression in ccRCC compared to normal tissue (1218). However, we found both to be undetectable in whole blood by qPCR. Several other candidate genes were found to be undetectable in whole blood, while most of the genes that were detectable (EGLN3, CAV2, ESM1, TMEM45A, NOL3, FABP7) did not exhibit significant dysregulation in expression between cancer and healthy PAXgene samples. A plausible path to overcoming this outcome was to examine these genes in plasma, as the mRNA levels (supposedly originating from the expression in blood cells) could significantly drop in healthy samples compared to cancer ones once the blood cells are removed, revealing the effect of the tumor derived RNA. However, after testing 10 plasma samples, our conclusion was that gene expression in the plasma was not measurable. The PAXgene system is used for stabilization and isolation of mRNA and other classes of nucleic acids (such as genomic DNA and miRNA). Blood specimens are collected in tubes containing a stabilization reagent preventing nuclease degradation and transcriptional changes in anticoagulated whole blood, and stabilizing RNA for up to 3 days at room temperature, for the purpose of expression profiling [51]. All handling of RNA was done with special care, and although it is reasonable to assume that for many or all of the candidate genes RNA was degraded by blood RNAses, the RNA integrity of whole PAXgene samples was indeed satisfactory, reflected by their high RIN values. Apart from the issues regarding RNA stability and concerning expression interference from blood cells, possible limitations of this work design may stem from the bioinformatics phase. Acquired GEO datasets, which were used to screen for genes absent from blood (with supposedly no blood expression) may not be 100% reliable; they came from many different sources and were not in a perfect mutual accordance. A separate issue is the cutoff value of < 1 rpkm to signify absence of blood presence for a gene. Most authors somewhat arbitrarily define the expression threshold as 1 rpkm (more generally anywhere between 0.3 rpkm and 1 rpkm), below which the sensitivity of RNA sequencing is insufficient to confirm expression and distinguish it from the background [52, 53]. Our cutoff potentially may have enabled genes with minute expression in blood cells to be included in the wet lab analysis.

### CDK18, CCND1, lox

The study also revealed the downregulation of two genes, CDK18 and CCND1, in ccRCC blood compared to healthy samples, as well as a tendency towards upregulation for LOX in metastatic compared to non-metastatic ccRCC. These results may be suitable for additional analysis in a larger patient cohort.

Cyclin-dependent kinase 18 (CDK18, PCTK3, PCTAIRE, PCTAIRE3) belongs to the PCTAIRE protein kinases, which are a subfamily of cdc2-related serine/threonine protein kinases named for a cysteine-for-serine substitution in the PCTAIRE motif conserved in the initially characterized CDK proteins (PCTAIRE sequence instead of the PSTAIRE sequence). They have unique N and C-terminal domains that extend forth from a serine/threonine kinase domain that is highly homologous to cdc2 [54]. PCTAIRE kinase subfamily includes three members, PCTK1/CDK16, PCTK2/CDK17, and PCTK3/CDK18 which are poorly researched. Insights into the activation of CDK18 have recently been obtained- it binds cyclin A2 and cyclin E1 (pulldown experiment with HEK293T cells) and is activated by cyclin A2 and PKA (cAMP-dependent protein kinase) [55]. CDK18 has recently been shown to regulate cell migration and adhesion in HEK293T cells by negatively modulating FAK (focal adhesion kinase) activity and reorganizing actin and associated skeletal/adhesion proteins such as cofilin, and has also been implicated in vesicular transport via interaction with Sec23Ap [56]. Overexpression of CDK18 also led to the formation of filopodia during the early stages of cell adhesion in HeLa cells [57]. Interestingly, it has also been recently found to play a role in replication stress and positively regulate genome stability, by associating with RAD proteins [58]. Lastly, PCTAIRE-3 as well as PCTAIRE-2 have been implicated in Alzheimer’s disease [59, 60]. CDK18 was induced by CTS-1 (Chimeric tumor suppressor-1, p53-derived synthetic tumor suppressor) and mediated growth arrest and death in glioma cells [61]. Apart from its activation by cyclin A2, in the same study CDK18 was shown to phosphorylate retinoblastoma tumorsupressor protein (Rb) in vitro [55]*. Although PCTAIRE1 has been found to be upregulated in many cancers, so far there is no such data for CDK18.*

Cyclin D1 (CCND1) regulates CDK4 or CDK6, whose activity is necessary for G1/S transition of the cell cycle. CCND1 is more frequently dysregulated in human cancers and therefore more studied than cyclin D2 or D3. Its overexpression leads to aberrant CDK activation resulting in rapid growth and division and is correlated with tumor stage, increased metastasis and poor prognosis in various cancers [62]. It is also involved in processes such as DNA repair and the control of mitochondrial activity and cell migration; it may assume CDK-independent functions as well [63]. CCND1 was investigated by microarray and TMA in ccRCC, and found to be upregulated and a potential therapeutic target [64]. In another study, CCND1 was found to be a useful immunohistochemical marker to discriminate between chromophobe renal cell carcinoma and renal oncocytoma [65].

Lysyl oxidase (LOX) performs covalent cross linking in elastin and collagen by oxidizing lysine residues, and therefore is important for the integrity of extracellular matrix [66]. It has both intracellular and extracellular functions and is involved in a number of pathological processes that affect the connective tissue [67]. It is upregulated in many cancers and involved in tumor progression, although it has been reported to function as a tumor-suppressor as well. Its concrete roles in various aspects of tumorigenesis have been reviewed recently [68]. LOX is a HIF target [69] and in ccRCC, LOX has been shown to be strongly overexpressed compared to normal tissue; it is one of the genes postranscriptionally regulated by miR-141-3p and miR-145-5p; and has prognostic relevance for the overall survival of ccRCC patients [70]. In ccRCC cell cultures, it has been found to function in a positive-regulative loop with HIF-1α, and to influence ccRCC progression by modifying cellular adhesion, migration, and the rigidity of the collagen matrix [71].

## Conclusions

In summary, with the goal of finding potential blood-based biomarkers for ccRCC, this study investigated the blood presence of genes highly overexpressed in ccRCC tissue and compared their blood mRNA levels between healthy and ccRCC patient samples. The overexpression in tissue was not reflected in the increase in the levels of mRNAs circulating in the blood of ccRCC patients. The analysis revealed the transcripts of CDK18 and CCND1 as underexpressed in the blood of ccRCC patients, and LOX as showing a tendency towards upregulation in metastatic ccRCC compared to non-metastatic. Further analysis of the selected gene panel by using a larger patient cohort may prove useful.

## Supplementary information

**Additional file 1:.** A subset of genes were detected using UPL probes in which case probe numbers are given.

**Additional file 2:** All cancer samples (metastatic and non-metastatic) were included in a single group and compared with normal samples.

**Additional file 3:** Metastatic cancer samples were taken as a separate group and compared to non-metastatic.

## Data Availability

All the data are available from the corresponding author upon request.

## Abbreviations

CCND1: Cyclin d1
ccRCC: Clear cell renal cell carcinoma
CDK18: Cyclin-dependent kinase 18
CEA: Carcinoembryonic antigen
cfDNA: Circulating cell-free dna
circRNA: Circular rna
CNV: Copy number variation
CTCs: Circulating tumor cells
GEO: Gene expression omnibus database
HCC: Hepatocellular carcinoma
HIF: Hypoxia inducible factor
lncRNA: Long non-coding rnas
LOX: Lysyl oxidase
miRNAs: Micrornas
PSA: Prostate-specific antigen
RCC: Renal cell carcinoma
RT-qPCR: Quantitative real time pcr
TCGA: Cancer Genome Atlas database
TiGER: Tissue-specific Gene Expression and Regulation database
